# Sulfated Nutrition Modifies Nutrient Content and Photosynthetic Pigment Concentration in Cabbage under Salt Stress

**DOI:** 10.3390/plants13101337

**Published:** 2024-05-13

**Authors:** Fresia Pacheco-Sangerman, Fernando Carlos Gómez-Merino, María Guadalupe Peralta-Sánchez, Libia I. Trejo-Téllez

**Affiliations:** 1 Programa de Edafología, Colegio de Postgraduados Campus Montecillo, Carretera México-Texcoco km 36.5, Montecillo C. P. 56264, Estado de México, Mexico; pacheco.fresia@colpos.mx (F.P.-S.); mgperalta@colpos.mx (M.G.P.-S.); 2 Programa de Recursos Genéticos y Productividad-Fisiología Vegetal, Colegio de Postgraduados Campus Montecillo, Carretera México-Texcoco km 36.5, Montecillo C. P. 56264, Estado de México, Mexico; fernandg@colpos.mx

**Keywords:** Brassicaceae, *Brassica oleracea*, S metabolism, abiotic stress, salinity, nutrient status

## Abstract

Negative effects of salt stress may be counteracted by adequate management of sulfated nutrition. Herein, we applied 3.50, 4.25, and 5.00 mM SO_4_^2−^ in a nutrient solution to counteract salt stress induced by 75 and 150 mM NaCl in cabbage cv. Royal. The increase in NaCl concentration from 75 to 150 mM reduced the contents of macronutrients and micronutrients in the shoot. When increasing from 3.50 to 4.25 mM SO_4_^2−^, the contents of nitrogen (N), phosphorous (P), potassium (K), calcium (Ca), magnesium (Mg), and sulfur (S) in shoots were enhanced, at both concentrations of NaCl. Increasing from 3.50 to 4.25 mM SO_4_^2−^ enhanced iron (Fe), zinc (Zn), manganese (Mn), and sodium (Na) concentrations with 75 mM NaCl. With 150 mM NaCl, the increase from 3.50 to 4.25 mM SO_4_^2−^ enhanced the contents of Cu and Mn, but also those of Na. Chlorophylls a, b, and total decreased as the concentration of SO_4_^2−^ increased in plants treated with 150 mM NaCl. With 75 mM NaCl, carotenoid concentration had a positive relationship with SO_4_^2−^. Hence, the 4.25 mM SO_4_^2−^ concentration increased the contents of macronutrients and micronutrients in the presence of 75 mM NaCl, while, with 150 mM NaCl, it improved the contents of macronutrients except K. The chlorophyll a/chlorophyll b ratio remained close to 3 when the plants were treated with 5.00 mM SO_4_^2−^, regardless of NaCl. Similarly, this level of SO_4_^2−^ increased the concentration of carotenoids, which translated into reductions in the total chlorophyll/carotenoid ratios, indicating a protective effect of the photosynthetic apparatus. It is concluded that higher doses of sulfur favor the accumulation of nutrients and increase the concentration of carotenoids under salt stress.

## 1. Introduction

As a consequence of climate change, plants experience various types of abiotic stresses, including high-energy radiation, high and low temperatures, floods, droughts, and salinity [1], which limit the production of economically important crops by reducing yields by up to 70% [2]. It is estimated that salinity affects about 1125 million hectares of soil in the world, and, each year, 1.5 million hectares are added to the list of soils considered unsuitable for agriculture because of high salt concentrations [3].

Salinity causes osmotic stress due to a reduction in water availability resulting from high concentrations of solutes in the medium, subsequently generating ionic stress due to an excessive accumulation of Na^+^ and Cl^−^, and oxidative stress that damages proteins, membranes, and nucleic acids [4]. The most affected process is photosynthesis since the thylakoid membranes where the chlorophylls are anchored are altered, affecting photosystem II, the oxygen evolution complex, electron transport, chlorophyll biosynthesis, and the synthesis of ATP and NADPH [5,6]. As photosynthetic and respiratory processes are affected, plant cells generate reactive oxygen species (ROS) that cause oxidative stress [7].

From a nutritional point of view, salinity alters the balance of cations and anions [8]. Under these conditions, the absorption and translocation of NO_3_^−^ [9] is restricted since Cl^−^ competes with NO_3_^−^ and causes toxicities and ionic imbalances. An increase in Na^+^ influx causes lower K^+^ uptake and transport [10] and a decrease in H_2_PO_4_^−^, Ca^2+^, and Mg^2+^ contents. Consequently, the photosynthetic growth rate and yield decrease.

To counteract the negative effects of salt stress, plants trigger various adaptation mechanisms, for example, changes in the expression and regulation of genes allow them to adapt their morphology, physiology, and biochemistry in response to salinity [11].

Among the mechanisms of tolerance to salinity are the following: the regulation of osmotic balance through the accumulation of compatible solutes, such as proline, soluble sugars, glycine betaine, and polyols; homeostasis and compartmentalization of ions to avoid ionic toxicities; sodium exclusion from roots; activation of Na^+^/H^+^ antiporters (which counteract the accumulation of Na^+^ and reduces its negative effect on the cytosol); salt secretion by glands, leaf succulence, and protection of photosynthesis; and reduction of water loss in shoots [11,12,13]. Specifically, to deal with oxidative stress, plants synthesize antioxidants that are divided into enzymatic components, such as superoxide dismutase (SOD), catalase (CAT), ascorbate peroxidase (APX), guaiacol peroxidase (GP), glutathione reductase (GR), monodehydroascorbate reductase (MDAR), and dehydroascorbate reductase (DHAR), and non-enzymatic antioxidants, such as acid ascorbic, reduced glutathione, α-tocopherol, carotenoids, flavonoids, and proline [14].

Some families of economically important plants, such as the Brassicaceae, have developed mechanisms to reduce the damage caused by salt stress, through the accumulation of organic and inorganic osmolytes [15] and the synthesis of glucosinolates that contain structural S [16]. From S, plants synthesize cysteine, glutathione, thioredoxins, thiamine, biotin, CoA, S-adenosyl-methionine, Fe-S cluster, methionine, and its derivatives, which help in terms of tolerance to salinity [17]. S also participates in cell membrane formation, electron transport, photosynthesis, and N assimilation [18]. In this research, the effect of applying increasing doses of SO_4_^2−^ to counteract salt stress in cabbage (*Brassica oleracea* L. var. *capitata*) plants was evaluated, considering as response variables the contents of macronutrients and micronutrients in shoots, as well as of leaf concentrations of photosynthetic pigments (chlorophylls and carotenoids).

Herewith, we aimed to evaluate the effect of sulfur on the tolerance of cabbage (*Brassica oleraceae*) cv. Royal to salt stress by measuring nutritional content and concentrations of photosynthetic pigments. It is hypothesized that the increase in sulfate concentration in the nutrient solution could mitigate the negative effects of salinity in cabbage.

## 2. Results

### 2.1. Nutrient and Sodium Content

We determined the contents of macronutrients and micronutrients in order to elucidate the effects of different levels of sulfate added to the nutrient solution on the uptake and accumulation of the essential elements in the shoots of cabbage plants exposed to salt stress.

**Figure 1:**
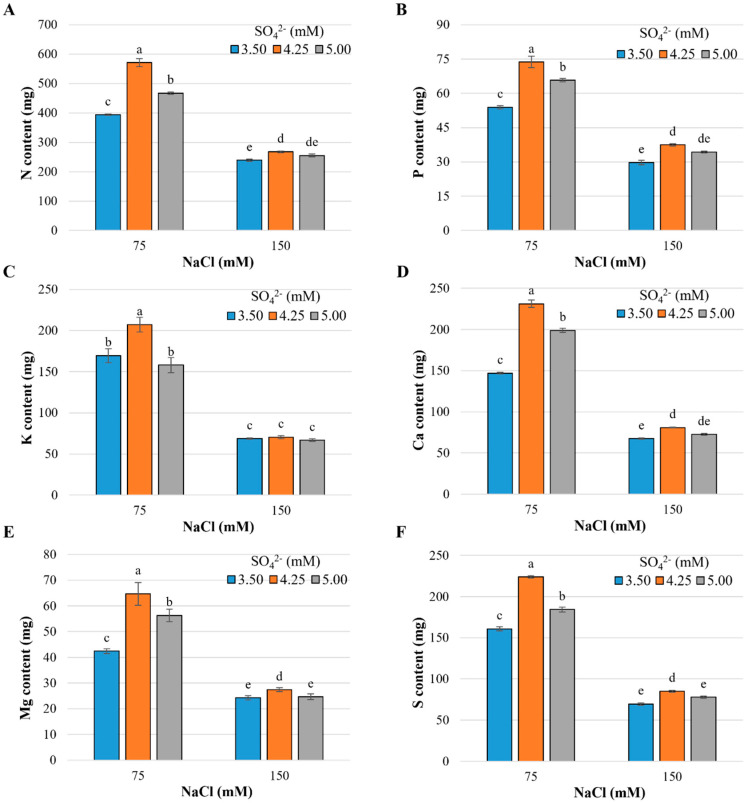
Macronutrient content in shoots of cabbage cv. Royal plants exposed to salt stress induced by sodium chloride and different doses of sulfate. (**A**) Nitrogen, (**B**) Phosphorous, (**C**) Potassium, (**D**) Calcium, (**E**) Magnesium, (**F**) Sulfur. Means ± SD with different letters in each subfigure indicate statistically significant differences among treatments (Tukey, *p* ≤ 0.05). n = 5.

Nutrient and sodium contents were significantly affected by both the main effects of the study factors (Tables S1 and S2) and their interaction. In this study, only the interaction results are reported. The increase in the NaCl concentration from 75 to 150 mM in the nutrient solution reduced the macronutrient contents in shoots. Likewise, differential effects of SO_4_^2−^ concentrations in the nutrient solution were observed, which were dependent on the salinity level (Figure 1).

The contents of N, P, K, Ca, Mg, and S were in the following ranges: 239.2–570.9, 29.7–73.7, 66.9–207.2, 67.5–231.3, 64.7–24.3, and 223.9–69.6 mg, respectively. Except for the K contents, which were the lowest contents recorded in the treatment, at 3.5 mM SO_4_^2−^ and 150 mM NaCl. The highest contents of all macronutrients were observed in plants treated with 4.25 mM SO_4_^2−^ in the presence of 75 mM NaCl (Figure 1).

The increase from 3.50 to 4.25 mM SO_4_^2−^ in the nutrient solution increased the N contents by 44.9 and 11.9% (Figure 1A), the P contents by 36.7 and 26% (Figure 1B), the Ca^2+^ contents by 57.4 and 19.9% (Figure 1D), those of Mg^2+^ by 52.5 and 12.9% (Figure 1E), and those of S by 39.2 and 22.2% (Figure 1F), with 75 and 150 mM NaCl, respectively. In the case of K^+^ content, the positive impact of increasing from 3.50 to 4.25 mM SO_4_^2−^ was only observed with the 75 mM NaCl dose, with increases of 22.2% (Figure 1C).

On the other hand, when increasing from 3.50 to 5.00 mM SO_4_^2−^, only with the low dose of NaCl (75 mM) were increases recorded in N content of 18.6% (Figure 1A), P of 21.9% (Figure 1B), Ca^2+^ of 35.5% (Figure 1D), Mg^2+^ of 32.7% (Figure 1E), and S of 14.5% (Figure 1F).

**Figure 2:**
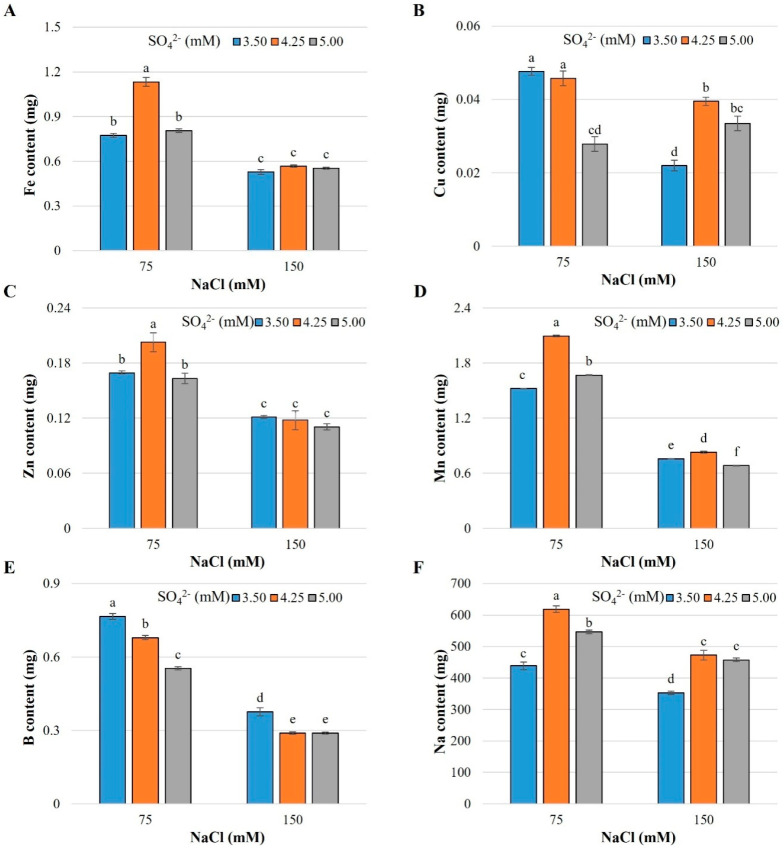
Micronutrient and sodium content in shoots of cabbage cv. Royal plants exposed to salt stress induced by sodium chloride and different doses of sulfate. (**A**) Iron, (**B**) Copper, (**C**) Zinc, (**D**) Manganese, (**E**) Boron, (**F**) Sodium. Means ± SD with different letters in each subfigure indicate statistically significant differences among treatments (Tukey, *p* ≤ 0.05). n = 5.

Regarding micronutrients, the contents of Fe ranged from 0.53 to 1.13 mg; while the content ranges of Cu, Zn, Mn, and B were 0.02–0.05, 0.11–0.21, 0.68–2.10, and 0.29–0.77 mg, respectively (Figure 2).

The increase in the NaCl concentration from 75 to 150 mM reduced the micronutrient and sodium contents in the shoot. The SO_4_^2−^ levels evaluated caused differential effects depending on the dose of NaCl (Figure 2). With the 75 mM NaCl dose, it was observed that increasing the dose of SO_4_^2−^ from 3.50 to 4.25 mM increased the Fe^2+^ content by 46.5% (Figure 2A), that of Zn^2+^ by 19.9% (Figure 2C), that of Mn^2+^ by 37.7% (Figure 2D), and that of Na by 40.8% (Figure 2F). Similarly, increasing SO_4_^2−^ from 3.50 to 4.25 mM in the nutrient solution increased the contents of Cu^2+^, Mn^2+^, and Na^+^ in the shoot by 79.7, 9.5, and 33.8%, respectively, with the dose of 150 mM NaCl (Figure 2B,D,F). On the other hand, the B content in the shoot decreased significantly with the increase in both NaCl and SO_4_^2−^ in the nutrient solution (Figure 2E).

### 2.2. Photosynthetic Pigments

The photosynthetic pigments analyzed in leaves were chlorophylls a and b, total chlorophyll, and carotenoids. With their concentration values, the chlorophyll a/chlorophoryl b and total chlorophyll/carotenoid ratios were estimated.

The interaction of the study factors was significant in the concentrations of photosynthetic pigments; therefore, only their results will be presented.

**Figure 3:**
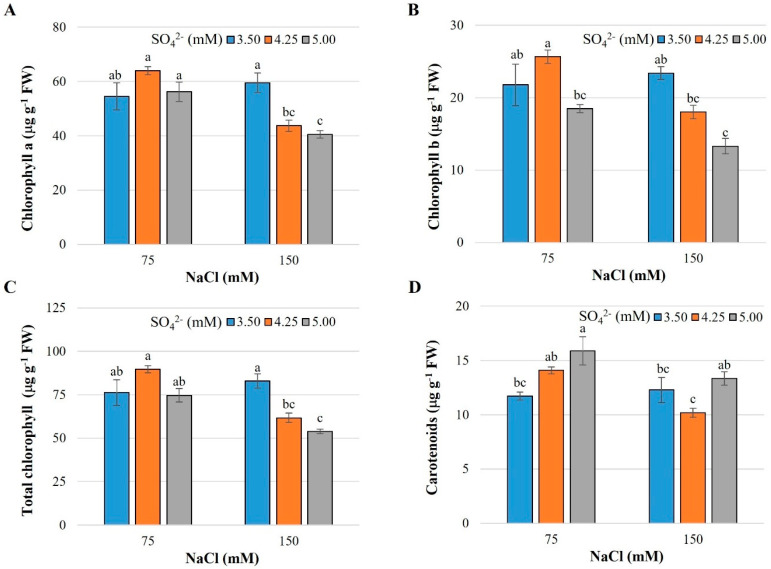
Concentration of photosynthetic pigments in leaves of cabbage cv. Royal plants exposed to salt stress induced by sodium chloride and different doses of sulfate. (**A**) Chlorophyll a, (**B**) Chlorophyll b, (**C**) Total chlorophyll, (**D**) Carotenoids. Means ± SD with different letters in each subfigure indicate statistically significant differences among treatments (Tukey, *p* ≤ 0.05). n = 5.

The concentrations of chlorophylls a, b, and total decreased as the concentration of SO_4_^2−^ increased when the plants were treated with 150 mM NaCl (Figure 3A–C). On the contrary, when the plants were exposed to 75 mM NaCl, increasing the SO_4_^2−^ dose from 3.50 to 5.00 mM increased the carotenoid concentration by 35.5% (Figure 3D).

The chlorophyll a/chlorophyll b ratio was higher in the treatments with the 5.00 mM SO_4_^2−^ dose with both doses of NaCl. On the contrary, in these same treatments, the total chlorophyll/carotenoid ratio was reduced (Table 1).

## 3. Discussion

Salinity causes osmotic stress in plants due to high concentrations of solutes in the growth medium, which reduce the availability of water in the roots and induce ionic stress as a consequence of the excessive accumulation of Na^+^ and Cl^−^. Moreover, oxidative stress occurs due to the alteration of the electron transport chain. Additionally, nutritional stress occurs due to an imbalance in the absorption, translocation, and assimilation of essential elements [19]. The results obtained in this study showed that the level of 150 mM NaCl considerably reduced the concentration of macronutrients (Figure 1), which is associated with the antagonistic effects between Na^+^ and Cl^−^ with nutrients. In general, the absorption and assimilation of N, P, K^+^, Ca^2+^, Mg^2+^, and S decrease with increasing salinity levels [20].

By decreasing N absorption, the formation of amino acids, amides and proteins, polyamines, and quaternary compounds is reduced [21]. N is a determining factor in the synthesis of chlorophylls, amino acids, proteins, nucleic acids, enzymes, plant hormones, and osmolytes, which participate in the mechanisms of tolerance to abiotic stress [22]. Because of this, it is important to maintain adequate N concentrations when plants grow under salt-stress conditions.

The decrease in N content (Figure 1A) is mainly due to the competition between NO_3_^−^ and Cl^−^ absorption channels, since the latter uses anion transporters including nitrate [23], but also phosphate [24] and sulfate [25,26], which also reduces the P and S contents in plants (Figure 1B,F). As an essential element, chlorine participates in the regulation of enzymatic activities in the cytosol, is a cofactor of the photosynthetic complex in water photolysis, and has an important function in cell turgor and pH [27]. However, when applied in high doses, this essential element decreases the transport of NO_3_^−^, which causes an increase in its translocation to the aerial part, affecting cellular homeostasis. Excess Cl^−^ leads to the entry of this ion into the interior of chloroplasts and mitochondria, compartments in which it alters electron transport chains and promotes the formation of ROS [23]. The above alters metabolic processes, especially when the plant does not have an efficient antioxidant system that eliminates the overproduction of ROS.

In the same way, the Mg^2+^ content was reduced in cabbage plants with the 150 mM NaCl concentration (Figure 1E) because the simultaneous movement of Cl^−^ and Na^+^ has an antagonistic effect with Mg^2+^, causing a reduction in its absorption rate [28].

On the other hand, the negative effects of salinity on the absorption and accumulation of essential elements, such as K^+^ and Ca^2+^, are due to the fact that, under normal conditions, plants maintain a membrane potential difference of around -140 mV, which changes under salt stress conditions. In particular, the increase in Na^+^ concentration depolarizes the membrane, and a new electrochemical gradient is established that favors the passive transport of Na^+^ towards the cytosol [29], affecting the K^+^ and Ca^2+^ transporters which are voltage-dependent. The increase in the concentration of Na^+^ in the cell gives way to an interaction between Na^+^ ions and the carbonyl groups of lipids [30], forming Na^+^–lipid complexes with reduced mobility in the cell membrane [31]. Phosphatidylcholine lipids show stronger interactions with Na^+^ with respect to phosphatidylethanolamine lipids since hydrogen bonds are established with the latter, both intramolecular and intermolecular, forming a more densely packed bilayer [32].

As a consequence of the aforementioned interactions, changes occur in the composition of membrane lipids and the activity of proteins is altered, decreasing their permeability and selectivity for water, ions, and metabolites [33]. This explains the low capacity of the cell to prevent the massive entry of Na^+^ and Cl^−^. Another negative effect of Na^+^ on the membrane is the interaction it establishes with calcium. As a second messenger in cellular signal transduction, Ca^2+^ is essential in improving tolerance to abiotic stress [34]. In this study, it was observed that the application of 150 mM NaCl significantly reduced the Ca^2+^ content in the shoots (Figure 1D). The above causes the displacement of Ca^2+^ ions by Na^+^ ions, which destabilizes the lipid bilayer of the membrane and reduces its selectivity. In turn, that displacement increases membrane permeability and causes intracellular extravasation of cations and a high Na^+^/K^+^ ratio, which causes an undesirable ionic imbalance for the cell [35].

In the case of K^+^, the results showed that the highest concentration of NaCl drastically reduced its content in the plant (Figure 1C). The entry of Na^+^ through the plasma membrane in roots occurs mainly by voltage-independent non-selective cation channels (NSCCs) and high-affinity potassium transporters (HKTs and HAKs) [36]. Since both K^+^ and Na^+^ share the same transport mechanism, they both compete when the plant is under salt stress, causing a reduction in K^+^ uptake [37]. Na^+^ influx also depolarizes the membrane, activating outward rectifying K^+^ (GORK) channels, through which K^+^ is excluded from plant cells and tissues [38] causing an imbalance in the K^+^/Na^+^ ratio and reducing the absorption and accumulation of K^+^ in plants.

We observed that the 4.25 mM SO_4_^2−^ dose increased the content of all macronutrients (N, P, K^+^, Ca^2+^, Mg^2+^, and S) under conditions of moderate salt stress (75 mM NaCl). Sulfur improves the absorption of nutrients due to synergistic relationships with them [39]. Furthermore, under salt stress conditions, sulfur metabolites such as amino acids, vitamins, the thioredoxin system, glutathione lipoic acid, and glucosinolates have a high potential to promote or modify physiological and molecular processes in higher plants [40]. S-containing compounds play an important role in the response of plants to abiotic stress factors. S participates in the biosynthesis of cysteine and methionine, resistance against diseases, and detoxification of ROS, xenobiotics, and non-essential transition metals [41]. By increasing the dose to 5.00 mM SO_4_^2−^, the nutrient content in the shoot was reduced, probably because poor growth under salt stress decreases sulfate demand since there is a negative regulation of the transporters [42]. Cysteine, whose precursor is SO_4_^2−^, plays an important role in the synthesis of ABA (abscisic acid) and glutathione, a defense compound against stress. S metabolism and ABA biosynthesis interact to ensure sufficient cysteine for ABA production [43], which plays a central integrative function by activating an adaptive signaling cascade and regulating gene expression in response to salt stress [13].

Salinity affects plant development as it alters the availability of micronutrients and the competition for their absorption, assimilation, and transport [20]. In our study, we observed that the application of 150 mM NaCl reduced the Fe^2+^ content in plants (Figure 2A), which is associated with the inhibition of H^+^-ATPase activity in the root, which, in turn, reduces the acidification of the root apoplast and the rhizoplane zone. Additionally, the availability of Fe^2+^ is reduced at alkaline pH values and there is an antagonism between Na^+^ and Fe^2+^ [44]. This same trend is observed in the contents of Zn^2+^ (Figure 2C), Mn^2+^ (Figure 2D), and B (Figure 2E), whose contents decreased significantly with the 150 mM NaCl concentration. Nevertheless, it is important to highlight that the negative effect of the 75 mM NaCl salinity level on the Fe^2+^, Zn^2+^, and Mn^2+^ contents was mitigated with the dose of 4.25 mM SO_4_^2−^ (Figure 2A,C,D). With regard to the Cu^2+^ content in this study, although it also decreased with increasing salinity level, it was observed that the increases in the dose of SO_4_^2−^ to 4.25 and 5.00 mM were positive (Figure 2B). This indicates that the absorption of these micronutrients is constant at moderate concentrations of salt stress (75 mM NaCl) in cabbage. The availability of micronutrients depends on the solubility of the element, the pH, the redox potential, and the nature of the binding sites. At high pH values, the availability of Fe^2+^, Cu^2+^, Zn^2+^, and Mn^2+^ is reduced since the ionic forms are oxides and hydroxides [20].

Under salinity conditions, the absorption and assimilation of micronutrients are highly variable depending on the plant species, exposure time, and salinity level. For example, concentrations of Mn^2+^ and Zn^2+^ increase in shoots of barley (*Hordeum vulgare*), tomato (*Solanum lycopersicum* L.), and rice (*Oryza sativa* L.), and decrease in corn (*Zea mays* L.). Likewise, the concentration of Fe^2+^ in tomatoes and rice increases, while it decreases in corn and barley [20]. The stress induced by 150 mM NaCl decreased the concentrations of Fe^2+^, Mn^2+^, and Zn^2+^ in the roots and shoots of different rice cultivars [45], which coincides with the findings shown here.

The treatment consisting of 4.25 mM SO_4_^2−^ and 75 mM NaCl increased the contents of Fe^2+^, Zn^2+^, and Mn^2+^ with respect to the doses of 3.50 and 5.00 mM SO_4_^2−^ with the same level of salinity (Figure 2A,C,D). Likewise, the 4.25 mM SO_4_^2−^ dose with 150 mM NaCl increased the Cu^2+^ and Mn^2+^ contents with respect to the other two sulfate levels and the same NaCl concentration (Figure 2B,D).

In sunflower (*Helianthus annuus* L.) and mustard (*Brassica juncea* L.), S has been documented to significantly promote the absorption of B [46]; however, in this research work, negative effects of increasing the dose of SO_4_^2−^ in the nutrient solution on the B content were observed, with both levels of NaCl tested (Figure 2E).

Salt stress damages photosynthetic pigments by reducing intermediates of chlorophyll biosynthesis, such as protochlorophyllide, Mg-photoporphyrin-IX, and protoporphyrin-IX [47]. Furthermore, NaCl stress limits photosynthesis due to the inhibition of electron transport and inactivation of photosystem II (P680) reaction centers, destruction of the oxygen evolution complex, and blockage of PSII electron transfer from the primary quinone acceptor to the secondary quinone acceptor [6]. Salt stress also leads to low availability and fixation of CO_2_, due to stomatal closure, which can result in an imbalance between the excitation of electrons and their utilization by photosynthesis, leading to the production of ROS, especially the superoxide anion (O_2_^−^) and hydrogen peroxide (H_2_O_2_) [5].

The function of chlorophylls is of great importance since they capture solar energy and transform it into chemical energy (ATP and NADPH). Chlorophyll a is the most efficient photosynthetic pigment since the absorption spectrum of chlorophyll b is less suitable when it is not combined with that of chlorophyll a [48], and its main function is to protect chlorophyll a from excess light [49]. The ratio between chlorophyll a and chlorophyll b found in plants is 3:1 [50]. In this research, the ratios between chlorophyll a and chlorophyll b presented values of around 2.5 with doses of 3.50 and 4.25 mM SO_4_^2−^ under salinity conditions, both at 75 and 150 mM NaCl (Table 1). Coincidentally, reductions in this relationship were reported in the review by Ashraf and Harris [51] in three of four Brassica species and in wheat (*Triticum aestivum* L.) cultivars susceptible to water deficit. By increasing the supply of SO_4_^2−^ to 5.00 mM, in both salinity conditions, the ratio rose to 3.05 (Table 1), which indicates that sulfate mitigates the negative effects caused by salinity. The value of the chlorophyll a/chlorophyll b ratio is positively related to maximal fluorescence [52]. Additionally, the *hpe1* (*high photosynthetic efficiency 1*) gene has been identified by mutation breeding in *Arabidopsis thaliana* (L.) Heynh., which increases the chlorophyll a/chlorophyll b ratio as a mechanism to improve photosynthesis under stress conditions [53].

The total chlorophyll/carotenoid ratio was significantly reduced by supplying 5.00 mM SO_4_^2−^ under both salinity levels tested (Table 1). Grape (*Vitis vinifera* L.) leaves exposed to excessive sunlight transfer excess energy to other pigments, carotenoids, which can then release it as heat. This increase in the proportion of carotenoids in the pigment complex results in a reduction in total chlorophyll/carotenoids, confirming its photoprotective function [54].

When applying 75 mM NaCl, the SO_4_^2−^ levels had no influence on the concentration of chlorophyll a and total chlorophyll. Contrarily, with 150 mM NaCl, the concentrations of chlorophyll a, b, and total were inversely related to the concentration of SO_4_^2−^ (Figure 3A–C). Likewise, the concentration of chlorophyll b, with both NaCl levels tested, was reduced with the 5.00 mM SO_4_^2−^ dose (Figure 3B). A greater impact of S on chlorophyll biosynthesis was expected against the oxidative stress triggered by salinity, as a protective mechanism in chloroplasts. Within the antioxidant system of chloroplasts, there are thiol peroxidase enzymes of the peroxiredoxin and glutathione peroxidases, and ascorbate peroxidase types, which are the main H_2_O_2_ detoxifying enzymes. In *Arabidopsis thaliana*, a species that belongs to the Brassicaceae family, Fe-dependent superoxide dismutases (Fe-SOD), Cu-Zn-dependent superoxide dismutases (CuZn-SOD), and two types of ascorbate peroxidases (APX; a thylakoid-bound tAPX and a soluble sAPX) are active in chloroplasts [55]. This suggests efficient control between the production and elimination of ROS under salt-stress conditions that is reflected in the maintenance of chlorophyll contents since the increase in antioxidant capacity provides protection to the photosynthetic system. In rice plants, when S was not supplied, the photosynthetic apparatus was severely affected and total chlorophyll content was reduced by 49% due to a general decrease in the efficiency of PSII and PSI. Additionally, the Rubisco content decreased in S-deprived plants [56], demonstrating the importance of S in photosynthesis, especially under abiotic stress conditions.

In Chinese (*Brassica rapa* L. ssp. Pekinensis) and white (*B. oleracea* var. *capitata* cv. Varaždinski) cabbage, applications of 50 and 100 mM NaCl increased the concentrations of chlorophylls a and b, whereas the application of 200 mM NaCl caused a decrease in these. On the other hand, in kale (*B. oleracea*, var. *Acephala* cv. IJK9), treatment with 50, 100, and 200 mM NaCl had no effect on chlorophyll concentration [57]. The above indicates a difference in tolerance to salt stress among species of the Brassicaceae family.

Carotenoids are essential structural components of the antenna complex, which act as auxiliary light-harvesting pigments and photoprotectors of the photosynthetic apparatus against excess light; additionally, they have antioxidant activity due to their ability to donate electrons [58]. Salinity-tolerant crops show increased synthesis of carotenoids, suggesting a protective role of these compounds [59].

The results showed an increase in the concentration of carotenoids with the dose of 5.00 mM SO_4_^2−^ in combination with 75 mM NaCl (Figure 3D), indicating a positive effect of S on the synthesis of these pigments under moderate salinity and, therefore, an important antioxidant mechanism under salt stress conditions in cabbage. Different compounds containing S act directly as antioxidants or modulate the antioxidant defense system. Among these, glutathione is considered one of the powerful antioxidants and protectors against various stress factors [60]. S-containing antioxidant compounds may aid in salt-stress tolerance along with stimulation of carotenoid biosynthesis. In tomato plants exposed to NaCl, the concentration of carotenoids in fruits increased with the highest levels of salinity (120 and 160 mM); this is associated with a response mechanism of the plants to oxidative stress, synthesizing non-enzymatic antioxidant molecules, such as carotenoids, for ROS detoxification [59].

## 4. Materials and Methods

### 4.1. Experiment Location and Experimental Conditions

This research was carried out under greenhouse conditions in Montecillo, the State of Mexico, Mexico (19.4646° N, 98.9039° W, 2250 m elevation). During the experimental phase, the average daytime and nighttime temperatures were 31.7 and 15.1 °C. The relative humidity during the day was 30% and 87% at night. The duration of the photoperiod was, on average, 11.3 h, with an average light intensity of 437 µmol m^−2^ s^−1^.

### 4.2. Plant Material

Three cabbage (*Brassica oleracea* L. var. *capitata*) cv. Royal seedlings, 30 d old, were established in 12 L, white, plastic containers in a floating root hydroponic system equipped with an oxygenation system (15 min oxygenation periods every 3 h). An acclimatization period of 14 d was established, during which the plants were irrigated with 50% Steiner [61] nutrient solution. The concentrations of macronutrients in the nutrient solution in mol_c_ m^−3^ were 6 NO_3_^−^, 0.5 H_2_PO_4_^−^, 3.5 SO_4_^2−^, 3.5 K^+^, 4.5 Ca^2+^, and 2 Mg^2+^, and those of micronutrients, in μM, were 90 Fe^2+^, 42 Mn^2+^, 7 Zn^2+^, 3 Cu^2+^, 40 B, and 1.8 Mo^2+^. The pH of the nutrient solution was adjusted to 5.0 and was formulated using analytical grade reagents (Meyer, Mexico City, Mexico).

### 4.3. Treatments and Experimental Design

A 2 × 3 factorial experiment was established. The first study factor was salt stress induced by NaCl with two levels, 75 and 150 mM. The second study factor was SO_4_^2−^ with three levels, 3.50, 4.25, and 5.00 mM. The combination of the levels of each factor created six treatments. Each treatment had four replicates. They were arranged in a completely randomized experimental design. The experimental unit was a 12 L container with three cabbage plants.

The corresponding levels of NaCl and SO_4_^2−^ were added to the 100% Steiner nutrient solution (macronutrients in mol_c_ m^−3^: 12 NO_3_^−^, 1 H_2_PO_4_^−^, 7 SO_4_^2−^, 7 K^+^, 9 Ca^2+^, and 4 Mg^2+^; and micronutrients in μM: 90 Fe^2+^, 42 Mn^2+^, 7 Zn^2+^, 3 Cu^2+^, 40 B, and 1.8 Mo^2+^); therefore, they only varied in the concentrations of SO_4_^2−^ and NaCl. The application of treatments began 14 days after transplantation. The pH of the initial solutions was 5.5 and was adjusted to this same value every 24 h. The nutrient solutions were renewed every 14 d and the application of treatments lasted 40 d.

### 4.4. Evaluated Variables

#### 4.4.1. Nutrient and Sodium Content

After 40 d of treatment, the concentrations of N, P, K^+^, Ca^2+^, Mg^2+^, S, Fe^2+^, Cu^2+^, Zn^2+^, Mn^2+^, B, and Na^+^ in the stems were determined.

N was determined by the micro-Kjeldahl method [62]. To do this, samples of 0.250 g of dry and ground shoot material were weighed and transferred to tubes, and then 2 mL of a bi-acid mixture [H_2_SO_4_:HClO_4_, 2:1, *v*:*v* (J. T. Baker, Phillipsburg, NJ, USA)] and 1 mL of H_2_O_2_ (J. T. Baker) were added. They were subsequently placed in a digestion plate at 300 °C. The samples were then removed and allowed to cool. Each sample was made up to 25 mL and filtered with deionized water in a previously labeled plastic container. From the resulting extract, 10 mL was taken for the determination of the N concentration.

For the determination of P, K^+^, Ca^2+^, Mg^2+^, S, Fe^2+^, Cu^2+^, Zn^2+^, Mn^2+^, B, and Na^+^, 0.5 g of each shoot sample was weighed in tubes and a biacid mixture [HNO_3_:HClO_4_, 2:1, *v*:*v* (J. T. Baker)] was added. They were subsequently placed in a digestion plate at 160 °C until an approximate volume of 2 mL was obtained. They were then made up to 25 mL with deionized water and filtered [63]. The resulting extracts were read on the inductively coupled plasma optical emission spectroscopy equipment (Agilent, ICP-Optical Emission Spectrometer, 725-ES, Santa Barbara, CA, USA).

With the dry shoot biomass data and the nutrient and sodium concentrations obtained, the contents of these elements were estimated.

#### 4.4.2. Photosynthetic Pigments

Leaf concentrations of chlorophylls a, b, and total, as well as carotenoids, were determined after 40 d of treatment. To do this, 0.5 g of leaf material was weighed, macerated with liquid N, and subsequently the mixture was homogenized with 10 mL of 80% acetone (J. T. Baker) [64]. The homogenized samples were centrifuged at 10,000 rpm for 15 min. The supernatant was separated into a clean tube and from there, 100 μL was taken and mixed with 900 μL of 80% acetone. Finally, the samples were read at the following wavelengths: 470, 646.8, and 663.2 nm in a spectrophotometer (JENWAY, 6715 UV/Vis; Leicestershire, UK). The blank used was 80% acetone. For the quantification of these pigments, the following equations were used:

$$Chlorophyll\;a=12.25A_{663.2}-2.79A_{646.8}$$

$$Chlorophyll\;b=21.54A_{646.8}-5.1A_{663.2}$$

$$Carotenoids=\frac{1000A_{470}-1.82C_{a}-85.02C_{b}}{198}$$

where: A is absorbance. The data were calculated in μg g^−1^ of fresh matter.

The ratios of chlorophyll a/chlorophyll b and total chlorophyll/carotenoid concentrations were calculated.

### 4.5. Statistical Analysis

The data obtained were statistically processed using the SAS 9.0 program. An analysis of variance was carried out for all measured variables. Likewise, a multiple comparison of means was carried out using the Tukey test (*p* ≤ 0.05).

## 5. Conclusions

It is concluded that the 4.25 mM SO_4_^2−^ concentration increased the contents of macronutrients and micronutrients, under conditions of moderate salt stress induced by the application of 75 mM NaCl. By increasing salt stress to 150 mM NaCl, the application of 4.25 mM SO_4_^2−^ improved the content of all macronutrients except K^+^. The increase in the concentration of SO_4_^2−^ from 3.5 to 4.25 mM increased the concentration of chlorophylls, only with 75 mM NaCl. Regarding the chlorophyll a/chlorophyll b ratio, it remained close to 3 when the plants were treated with 5.00 mM SO_4_^2−^, regardless of the dose of NaCl applied. Likewise, this level of SO_4_^2−^ increased the concentration of carotenoids, which reduced the total chlorophyll/carotenoid ratios, indicating a protective effect of the photosynthetic system. Therefore, higher doses of sulfur favor nutritional accumulation and increase the concentration of carotenoids under salt stress. These findings are significant since they confirmed the practical usefulness of increasing sulfate doses to improve the tolerance of cabbage to salt stress; likewise, they represent an alternative to the use of saline water in the production of other vegetables under hydroponic systems.

## Figures and Tables

**Table 1:** Chlorophyll a/chlorophyll b, and total chlorophyll/carotenoid ratios in leaves of cabbage cv. Royal plants exposed to salt stress induced by sodium chloride and different doses of sulfate.

NaCl (mM)	SO_4_^2−^ (mM)	Chlorophyll a/ Chlorophyll b Ratio	Total Chlorophyll/ Carotenoid Ratio
75	3.50	2.505	6.514
	4.25	2.500	6.364
	5.00	3.043	4.709
150	3.50	2.543	6.751
	4.25	2.426	6.057
	5.00	3.048	4.040

## Data Availability

The data presented in this study are available on request from the corresponding author.

## Supplementary Materials

The following supporting information can be downloaded at: https://www.mdpi.com/article/10.3390/plants13101337/s1, Table S1. Main effects of salt stress induced by sodium chloride and of different sulfate doses on macronutrient contents in shoots of cabbage cv. Royal plants. Table S2. Main effects of salt stress induced by sodium chloride and of different sulfate doses on micronutrient and sodium contents in shoots of cabbage cv. Royal plants.
