# Supplementary material for: Sulfated Nutrition Modifies Nutrient Content and Photosynthetic Pigment Concentration in Cabbage under Salt Stress
Source: Plants (Basel). 2024 May 13;13(10):1337. doi: 10.3390/plants13101337 (PMC11124958; doi:10.3390/plants13101337)
Supplement: Supplementary file 1 [file plants-13-01337-s001.zip › plants-2961936-supplementary.pdf]

Table S1. Main effects of salt stress induced by sodium chloride and of different sulfate doses on macronutrient contents in shoots of cabbage cv. Royal plants.

Study factor and levels	N	P	K	Ca	Mg	S
NaCl (mM)			(mg)			
75	477.41 ± 38.37 a	64.48 ± 4.45 a	178.28 ± 13.66 a	192.44 ± 18.20 a	54.46 ± 4.79 a	189.70 ± 13.65a
150	254.15 ± 7.14 b	33.87 ± 1.77 b	68.73 ± 1.55 b	73.73 ± 2.99 b	25.46 ± 0.80 b	77.52 ± 3.52 b
SO_4^{2-} (mM)						
3.50	316.64 ± 40.90 c	41.85 ± 6.43 c	119.19 ± 27.17 b	107.24 ± 20.94 c	33.35 ± 4.80 c	115.24 ± 24.14 c
4.25	419.40 ± 80.42 a	55.61 ± 9.71 a	138.86 ± 36.55 a	156.14 ± 39.72 a	46.04 ± 9.84 a	154.49 ± 36.62 a
5.00	361.31 ± 56.03 b	50.07± 8.29 b	112.46 ± 24.80 b	135.87 ± 33.36b	40.49 ± 8.34 b	131.10 ± 28.10 b

Means ± SD with different letters in each column and study factor indicate statistically significant differences among treatments (Tukey, $p \leq 0.05$). n = 15 for NaCl and n =10 for SO_4^{2-}.

Table S2. Main effects of salt stress induced by sodium chloride and of different sulfate doses on micronutrient and sodium contents in shoots of cabbage cv. Royal plants.

Study factor and levels	Fe	Cu	Zn	Mn	B	Na
NaCl (mM)			(mg)			
75	0.904 ± 0.086 a	0.040 ± 0.005 a	0.178 ± 0.011 a	1.762 ± 0.127 a	0.663 ± 0.046 a	534.638 ± 39.23 a
150	0.549 ± 0.014 b	0.031 ± 0.004 b	0.116 ± 0.006 b	0.756 ± 0.032 b	0.319 ± 0.023 b	427.641 ± 28.90 b
SO_4^{2-} (mM)						
3.50	0.651 ± 0.066 b	0.035 ± 0.007 b	0.14 5± 0.013 b	1.139 ± 0.202 c	0.571 ± 0.104 a	396.069 ± 24.36c
4.25	0.850 ± 0.151 a	0.043 ± 0.002 a	0.160 ± 0.024 a	1.462 ± 0.334 a	0.480 ± 0.101 b	545.435 ± 40.33 a
5.00	0.679 ± 0.067 b	0.031 ± 0.002 c	0.137 ± 0.015 b	1.176 ± 0.260 b	0.422 ± 0.070 c	501.917 ± 24.08 b

Means ± SD with different letters in each column and study factor indicate statistically significant differences among treatments (Tukey, $p \leq 0.05$). n = 15 for NaCl and n =10 for SO_4^{2-}.
